# Characterizing the Course of Loss of Control Eating and Prognostic Factors Following Bariatric Surgery: an Exploratory Analysis

**DOI:** 10.1007/s11695-023-06497-3

**Published:** 2023-02-18

**Authors:** Kathryn E. Smith, Aimee Varnado, J. Graham Thomas, Sivamainthan Vithiananthan, Daniel B. Jones, Pavlos Papasavas, Dale S. Bond

**Affiliations:** 1 Department of Psychiatry and Behavioral Sciences, University of Southern California, Los Angeles, CA 90033 USA; 2 Department of Psychiatry and Human Behavior, Weight Control and Diabetes Research Center, The Miriam Hospital/Brown Alpert Medical School, Providence, RI 02903 USA; 3 Department of Surgery, Cambridge Health Alliance, Cambridge, MA 02139 USA; 4 Department of Surgery, Rutgers New Jersey Medical School, Newark, NJ 07103 USA; 5 Department of Surgery, Hartford Hospital/HealthCare, Hartford, CT 06106 USA; 6 Department of Research, Hartford Hospital/HealthCare, Hartford, CT 06106 USA

**Keywords:** Bariatric surgery, Loss of control eating, Binge eating, Hedonic hunger, Ecological momentary assessment

## Abstract

**Purpose:**

Postoperative loss of control eating (LOCE) has detrimental associations with weight outcomes and mental health following bariatric surgery. However, little is known regarding LOCE course following surgery and preoperative factors that predict remittance, continuance, or development of LOCE. The present study aimed to characterize LOCE course in the year following surgery by identifying four groups: individuals with (1) postoperative de novo LOCE, (2) maintained LOCE (endorsed at pre- and post-surgery), (3) remitted LOCE (endorsed only at pre-surgery), and (4) those who never endorsed LOCE. Exploratory analyses examined group differences in baseline demographic and psychosocial factors.

**Materials and Methods:**

A total of 61 adult bariatric surgery patients completed questionnaires and ecological momentary assessment at pre-surgery and 3-, 6-, and 12-month postoperative follow-ups.

**Results:**

Results showed that 13 (21.3%) never endorsed LOCE prior to or after surgery, 12 (19.7%) developed LOCE after surgery, 7 (11.5%) evidenced remittance from LOCE after surgery, and 29 (47.5%) maintained LOCE prior to and after surgery. Relative to those who never endorsed LOCE, all groups who evidenced LOCE before and/or after surgery reported greater disinhibition; those who developed LOCE reported less planned eating; and those with maintained LOCE reported less satiety sensitivity and greater hedonic hunger.

**Conclusion:**

These findings highlight the importance of postoperative LOCE and need for longer-term follow-up studies. Results also suggest a need to examine the longer-term impact of satiety sensitivity and hedonic eating on LOCE maintenance, and the extent to which meal planning may buffer risk for de novo LOCE following surgery.

**Graphical Abstract:**

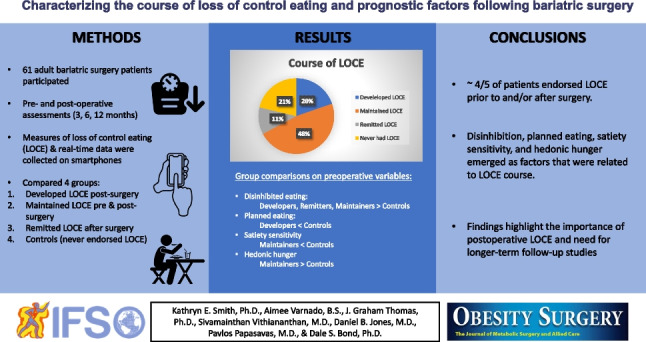

## Introduction

Loss of control eating (LOCE), which refers to the feeling that one cannot stop eating or control what or how much one is eating, is a maladaptive eating behavior that is a key feature of binge eating and the DSM-5 diagnosis of binge-eating disorder (BED) [1]. Both LOCE and BED are prevalent among candidates for bariatric surgery, with previous studies reporting approximately 40% of patients evidence LOCE prior to surgery [2, 3]. Furthermore, preoperative LOCE may persist following surgery, and despite evidence that LOCE and BED initially decrease after surgery, as time passes, the likelihood of recurrence or development of these behaviors increases [2–5]. Specifically, research has shown that LOCE is reported by 10–39% of patients within 2 years after surgery, though recurrent or de novo LOCE behavior can be present as early as six months post-surgery [3, 6]. Indeed, increasing evidence has shown a significant minority of patient evidence de novo LOCE, with one recent study finding that the prevalence of de novo BED and LOCE in the 7 years following surgery was approximately 5 and 25%, respectively [7].

Such findings are concerning given LOCE may have detrimental effects on bariatric surgery outcomes. While preoperative LOCE has not been a consistent predictor of weight outcomes, several studies have shown the presence of *postoperative* LOCE is related to suboptimal weight outcomes (i.e., less weight loss or greater weight regain) [4, 5, 8, 9]. Therefore, elucidating factors that are associated with the development or maintenance of LOCE after surgery is particularly relevant to improve weight outcomes. Moreover, LOCE can have negative implications that extend beyond poor weight outcomes, as binge eating and LOCE prior to and after surgery are associated with a range of negative psychosocial outcomes, including alcohol use disorder symptoms, lower self-esteem, and depressive symptoms [2, 7, 10]. As such, identifying and characterizing individuals who develop or maintain LOCE after surgery is particularly relevant for optimizing physical and mental health following surgery. Conversely, identifying factors that are associated with absence or remittance of LOCE may help to understand ways to mitigate negative outcomes.

Unfortunately, little is known regarding specific factors that increase or reduce risk for the development, continuation, or remittance of LOCE following surgery [11]. Binge-eating symptoms and obesity risk are strongly linked to negative emotionality (e.g., depression) [12, 13], hedonic hunger (i.e., preoccupation with and desire to consume foods for pleasure in the absence of physical hunger) [14], and disinhibition, which refers to the tendency to overeat in an obesogenic environment (e.g., eating in response to negative affect or food cues) [15]. Conversely, some traits and behaviors may have a positive influence on weight related changes and serve as protective factors against LOCE. For example, initiation of self-regulation behaviors is associated with better weight outcomes, including stopping eating when feeling full and stopping eating continuously during the day. These findings are supported by literature indicating that adaptive dietary restraint and satiety sensitivity (a component of interoception referring to the ability to perceive bodily signals of hunger and fullness) are key for self-regulation of food intake [16, 17]. In addition, self-monitoring of eating behavior and meal planning are core components of cognitive-behavioral and behavioral weight loss interventions, which have also been linked to decreased LOCE and better weight [7, 18].

Taken together, while it is known that LOCE is detrimental to patient health and that it mitigates the intended benefits of bariatric surgery, there is a dearth of research on the temporal course of postoperative LOCE and preoperative factors that contribute to change in this behavior following surgery. Understanding such factors has prognostic value given that a significant minority of patients develop or continue to engage in LOCE following bariatric surgery, and such data may help identify individuals who are most likely to experience negative effects of postoperative binge eating and LOCE. In turn, prevention and intervention efforts may be taken to remediate risk factors and/or increase protective factors. To address these gaps in the literature, the primary aim of the current study was to characterize the course of LOCE in the year following surgery by identifying four groups: individuals with (1) postoperative de novo LOCE, (2) maintained LOCE (i.e., endorsed at pre- and post-surgery), (3) remitted LOCE (i.e., endorsed only at pre-surgery), and (4) those who never endorse LOCE. As an exploratory aim, we also sought to examine differences in demographic and psychosocial factors between these groups at baseline. Based on prior research, psychosocial predictors included depressive symptoms, the proportion of eating episodes that were planned, and appetitive factors that relate to eating regulation (i.e., disinhibition, hedonic hunger, dietary restraint, and satiety sensitivity). It was expected that lower depressive symptoms, greater proportion of planned episodes, lower disinhibition and hedonic hunger, and higher restraint and satiety sensitivity would be related to a better LOCE course (i.e., absence or remittance of LOCE).

## Material and Methods

### Participants

The current study represents a secondary data analysis of a prospective cohort study examining psychosocial and behavioral predictors of bariatric surgery outcomes [19]. Participants were bariatric surgery patients recruited between May 2016 and April 2018 during clinic visits 3–8 weeks prior to surgery. Analyses were limited to participants who completed items from the Eating Disorder Examination Questionnaire (EDE-Q) [20] prior to surgery and at least one follow-up in order to be grouped according to LOCE status. The final analytic sample included 61 participants at pre-surgery, though there was attrition across the 3-month (*n* = 57), 6-month (*n* = 55), and 12-month follow-ups (*n* = 49).

### Procedure

The full protocol of the parent study was previously published and was registered at www.clinicaltrials.gov (NCT02777177) [19]. All procedures were approved by the institutional review board. Informed consent was obtained from all participants included in the study. Procedures that pertain to the current study are described as follows. After completing a phone screening, eligible patients attended a baseline visit at the research center that included the informed consent process, measurement of height and weight to calculate body mass index (BMI), and completion of questionnaires. Percent total weight loss (%TWL) was calculated at each assessment point as follows: ([baseline weight – follow-up weight (i.e., 3-, 6-, or 12-month weight)] ÷ baseline weight) × 100%. Participants also received training on the 10-day EMA protocol using a study-provided Android smartphone, which was administered using the PiLR Health™ application (MEI Research Ltd.). During the EMA protocol, participants received four semi-random prompts each day around the anchors of 11:00 am, 2:00 pm, 5:00 pm, and 8:00 pm. At each prompt, they were asked to respond to a series of questions (see “EMA Surverys”). Participants responded to approximately 10–60 questions per prompt, depending on whether certain behaviors (e.g., eating) were endorsed. Compliance was monitored by the EMA system and was viewable to participants in real-time. Participants were allowed to extend the EMA protocol if they experienced technical or other difficulties with the EMA protocol and were allowed additional days to provide data. After completing the EMA protocol, participants returned to the research center to receive compensation for their participation. Participants were compensated $75 for completing the baseline assessment, plus $0.50 for each completed EMA survey. Procedures were repeated at the 3-, 6-, and 12-month postoperative assessments.

### Questionnaire Measures

#### EDE-Q

Participants were administered selected items drawn from the EDE-Q, a widely-used self-report measure of eating psychopathology over the last 28 days [20]. Items included assessment of LOCE frequency (“Over the past 28 days, how many times did you have a sense of having lost control over your eating [at the time that you were eating])?” Participants were categorized into one of four groups based on LOCE status at baseline and follow-up as assessed by the EDE-Q: (1) those who never endorsed LOCE who served as a control group (CON); (2) those who endorsed at least one LOCE episode at one or more follow-up assessments but not prior to surgery (“developers” (DEV)); (3) those who endorsed at least one LOCE episode prior to surgery but not at any of their follow-up assessments (“remitters” (REM)); or (4) those who reported at least one LOCE episode prior to surgery and at least one follow-up assessment (“maintainers” (MAIN)).

#### Beck Depression Inventory (BDI-II)

Depressive symptoms were assessed using the BDI-II [21], which includes 21 items rated on a 4-point scale ranging from 0 to 3. Items are summed to compute a total score ranging from 0 to 63, with higher scores indicating more severe depressive symptoms.

#### EMA Surveys

Restraint and disinhibition were each assessed using five items adapted from the cognitive restraint (e.g., *I am conscious of what I eat*) and disinhibition (e.g., *When I feel upset, I overeat*) subscales of the Three Factor Eating Questionnaire [22]. Hedonic hunger was assessed using five items adapted from the power of food scale (e.g., *It’s very important to me that the foods I eat are as delicious as possible*) [23]. Each item was rated on a Likert‐type scale ranging from 1 (*never*) to 5 (*always*), with mean subscale scores calculated at each prompt. At each prompt, participants were also asked if they had eaten since the last prompt. If participants responded “yes,” satiety sensitivity was assessed by the item: *I stopped eating at the first sign of fullness* (yes/no). Participants were also asked whether or not (yes/no) the eating episode was planned (*I had planned to eat at this time*). Restraint, disinhibition, hedonic hunger, and satiety sensitivity were averaged across the 10-day protocol to create composite scores, and the proportion of planned eating episodes (number of planned eating episodes/number of eating episodes) across the 10-day EMA protocol was calculated.

### Statistical Analyses

Descriptive statistics were calculated for variables of interest. Generalized linear models (GLMs) and Chi-square tests were first used to compare groups on preoperative demographic factors. GLMs then assessed group status (CON, DEV, REM, or MAIN) as a predictor of each psychosocial variable of interest. Linear distributions were specified for all GLMs, with the exception of disinhibition, which evidenced a skewed distribution; as such, a gamma function was specified in this GLM. Covariates that were screened included age, sex, educational status, surgery type, preoperative BMI, %TWL, and race. Sex, surgery type, and preoperative BMI did not contribute significantly to any model and were subsequently removed in final analyses. A multivariate analysis of variance (MANOVA) also assessed the degree to which group status was related to %TWL at 3-, 6-, and 12-month follow-ups. Analyses were conducted using SPSS version 28.

## Results

Descriptive statistics are shown in Table 1. Of the final sample, 13 (21.3%) were categorized as CON, 12 (19.7%) as DEV, 7 (11.5%) as REM, and 29 (47.5%) as MAIN based on the EDE-Q. With respect to weight loss, the mean %TWL at 3, 6, and 12 months in this sample was 17.1%, 23.8%, and 27.1%, respectively. Group was not significantly associated with %TWL at any time point (*p*s = 0.138–0.799) and therefore was not included in the GLMs.

**Table 1:** Preoperative descriptive statistics

	Total (*n* = 61)		CON (*n* = 13)		DEV (*n* = 12)		REC (*n* = 7)		MAIN (*n* = 29)	
	M	SD	M	SD	M	SD	M	SD	M	SD
Age	44.39	11.54	48.62	11.72	42.33	13.00	45.57	13.55	43.07	10.42
BMI	45.85	7.01	45.96	8.46	46.49	6.34	44.44	3.33	45.87	7.48
BDI	8.39	7.30	8.00	9.75	5.08	5.33	8.71	4.42	9.86	7.15
Restraint	3.01	0.45	2.94	0.66	3.10	0.36	2.94	0.24	3.02	0.41
Disinhibition	2.37	0.83	1.74	0.36	2.20	0.96	2.62	0.50	2.67	0.83
Satiety sensitivity	0.71	0.33	0.90	0.22	0.74	0.26	0.72	0.41	0.61	0.35
Hedonic hunger	2.35	0.74	1.97	0.43	2.32	0.95	2.37	0.46	2.54	0.75
Planned eating (%)	81.35	22.15	0.91	0.16	0.72	0.29	0.80	0.24	0.81	0.21

*MAIN*, maintainer group; *REC*, recovered group; *DEV*, developer group; *CON*, control group; *BMI*, body mass index; *BDI*, Beck depression inventory-II. Planned eating reflects percentage of eating episodes that were planned

**Fig. 1:**
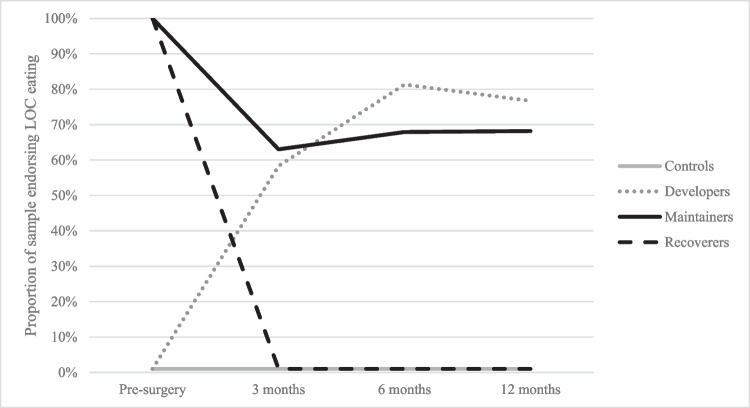
Loss of control eating (LOCE) before and after surgery across groups. Note: developers = individuals with postoperative de novo LOCE; maintainers = individuals with maintained LOCE (endorsed at pre- and post-surgery); remitters = individuals with remitted LOCE (endorsed only at pre-surgery); controls = individuals who never endorsed LOCE

There was also heterogeneity in the presence and timing of postoperative LOCE among those in the DEV and MAIN groups (Fig. 1). Among the DEV group, LOCE was endorsed by 58.3% of participants at 3 months, by 81.8% of participants at 6-months, and by 76.7% of participants at 12-months. In this group, 5 participants reported LOCE at all follow-ups, 2 reported LOCE at two follow-ups, and 5 reported LOCE at one follow-up. In the MAIN group, LOCE was endorsed by 63.0% of participants at 3 months, by 67.9% of participants at 6 months, and by 68.2% of participants at 12 months. In this group, 13 reported LOCE at one follow-up, 11 reported LOCE at two follow-ups, and 5 reported LOCE at all follow-ups.

### Comparison of Demographic Characteristics across LOCE Groups

Chi-square tests indicated that participant sex, race, marital status, preoperative BMI, and surgery type were not significantly associated with LOCE group status (sex: *χ*^2^ [1] = 2.89, *p* = 0.089; race: *χ*^2^ [1] = 0.04, *p* = 0.849; marital status: *χ*^2^ [1] = 0.02, *p* = 0.899; surgery type: *χ*^2^ [1] < 0.01, *p* = 0.959). Table 2 displays GLM results. Results of GLMs did not show LOCE group was related to preoperative BMI (Wald *χ*^2^ = 0.40, *p* = 0.941), age (Wald *χ*^2^ = 2.74, *p* = 0.434), or educational attainment (Wald *χ*^2^ = 2.03, *p* = 0.567).

**Table 2:** Tests of generalized linear model effects

	BDI		Restraint		Planned eating		Satiety sensitivity		Disinhibition		Hedonic hunger	
	Wald *χ*^2^	*p*	Wald *χ*^2^	*p*	Wald *χ*^2^	*p*	Wald *χ*^2^	*p*	Wald *χ*^2^	*p*	Wald *χ*^2^	*p*
Intercept	6.36	0.012	6.36	0.012	8.38	0.004	10.75	0.001	6.32	0.012	15.33	< .001
Educational attainment	3.16	0.076	3.16	0.076	7.65	0.006	0.14	0.711	0.05	0.827	1.63	0.202
Age	0.01	0.935	0.01	0.935	0.79	0.373	0.00	0.965	0.07	0.794	3.18	0.075
Race	1.51	0.681	1.51	0.681	1.56	0.669	1.84	0.607	8.19	0.042	2.30	0.513
LOCE group	3.74	0.291	3.74	0.291	5.40	0.145	**8.61**	**0.035**	**19.93**	** < .001**	**9.18**	**0.027**

*LOCE* loss of control eating. Bolded effects are significant at *p* < .05

### Comparison of Psychosocial Variables Across LOCE Groups

As shown in Table 2, there were not significant effects of group status as a predictor of BDI-II scores (Wald *χ*^2^ = 3.74, *p* = 0.291), restraint (Wald *χ*^2^ = 1.09, *p* = 0.780), or proportion of planned eating episodes (Wald *χ*^2^ = 5.397, *p* = 0.145); however, parameter estimates (Table 3) showed that the DEV group evidenced a lower proportion of planned episodes compared to the CON group (*B* =  − 0.19, *p* = 0.026). There were also significant group effects for satiety sensitivity (Wald *χ*^2^ = 8.61, *p* = 0.035), hedonic hunger (Wald *χ*^2^ = 9.18, *p* = 0.027), and disinhibition (Wald *χ*^2^ = 19.93, *p* < 0.001). Compared to the CON group, the MAIN group reported lower satiety sensitivity (*B* =  − 0.31, *p* = 0.004) and greater hedonic hunger (*B* = 0.69, *p* = 0.002). Regarding disinhibition, the MAIN, REM, and DEV groups reported greater levels compared to the CON group (MAIN: *B* = 0.45, *p* < 0.001, REM: *B* = 0.37, *p* = 0.009, DEV: *B* = 0.27, *p* = 0.034); no other group comparisons were statistically significant.

**Table 3:** Generalized linear model parameter estimates

	BDI			Restraint			Planned eating		
	*B*	*SE*	*p*	*B*	*SE*	*p*	*B*	*SE*	*p*
Intercept	14.76	5.83	0.011	3.09	0.35	< 0.001	0.51	0.17	0.003
Educational attainment	** − **1.38	0.78	0.076	0.03	0.05	0.569	0.06	0.02	0.006
Age	0.01	0.08	0.935	** − **0.01	0.01	0.354	0.00	0.00	0.373
Race (other)	** − **0.72	2.64	0.784	0.04	0.16	0.807	** − **0.07	0.08	0.368
Race (Hawaiian/Pacific Islander)	** − **9.41	8.03	0.241	0.03	0.49	0.960	0.17	0.23	0.474
Race (Black/African American)	0.07	2.13	0.973	** − **0.28	0.13	0.029	** − **0.03	0.06	0.664
LOCE group (MAIN)	2.34	2.37	0.323	0.03	0.14	0.825	** − **0.10	0.07	0.141
LOCE group (REC)	1.50	3.30	0.650	** − **0.01	0.20	0.968	** − **0.14	0.09	0.129
LOCE group (DEV)	** − **2.26	2.90	0.436	0.16	0.18	0.365	** − 0.19**	**0.08**	**0.026**
	Satiety sensitivity			Disinhibition			Hedonic hunger		
	*B*	*SE*	*p*	*B*	*SE*	*p*	*B*	*SE*	*p*
Intercept	0.79	0.27	0.003	0.55	0.26	0.036	1.81	0.56	0.001
Educational attainment	0.01	0.03	0.711	0.01	0.03	0.827	** − **0.10	0.07	0.202
Age	0.00	0.00	0.965	0.00	0.00	0.794	0.01	0.01	0.075
Race (other)	0.14	0.12	0.245	** − **0.25	0.12	0.032	** − **0.10	0.26	0.683
Race (Hawaiian/Pacific Islander)	0.28	0.36	0.435	** − **0.63	0.35	0.07	** − **1.13	0.78	0.147
Race (Black/African American)	0.06	0.10	0.571	** − **0.16	0.09	0.087	** − **0.13	0.21	0.527
LOCE group (MAIN)	** − 0.31**	**0.11**	**0.004**	**0.45**	**0.10**	** < .001**	**0.69**	**0.23**	**0.002**
LOCE group (REC)	** − **0.17	0.15	0.242	**0.37**	**0.14**	**0.009**	0.49	0.32	0.124
LOCE group (DEV)	** − **0.19	0.13	0.16	**0.27**	**0.13**	**0.034**	0.53	0.28	0.059

*BDI*, Beck depression inventory-II; *LOCE*, loss of control eating; *MAIN*, maintainer group; *REC*, recovered group; *DEV*, developer group. Race and LOCE group were coded such that White and Control (CON) were the reference categories. Bolded effects are significant at *p* < .05

## Discussion

The present study sought to characterize the course of LOCE in the first year after bariatric surgery and to explore potential differences in preoperative characteristics among groups who evidenced (1) postoperative de novo LOCE, (2) maintained LOCE across pre- and postoperative assessments, (3) remittance of LOCE following surgery, and (4) those who never endorse LOCE prior to or after surgery. In general, results indicated that a majority of patients (approximately two-thirds) reported some degree of LOCE during the initial postoperative year, though there was variability in the timing of LOCE. Although many patients evidenced maintained LOCE across pre- and post-surgery assessments, it is notable that approximately 20% of participants experienced de novo LOCE. These findings are consistent with prior research reporting a substantial number of de novo LOCE cases following bariatric surgery (7) and further highlight the importance of ongoing symptom monitoring to inform early intervention approaches. In addition, the course of LOCE was unrelated to surgery type, which aligns with prior research in this sample showing non-signficant relationships between surgery type and appetitive sensations [24].

While demographic characteristics did not associate with LOCE course, all groups who evidenced LOCE before and/or after surgery reported greater disinhibition compared to those who never endorsed LOCE. Such results are not surprising as studies consistently show associations between disinhibition and binge-eating symptoms, with some evidence indicating that disinhibition, LOCE, and binge eating are related components of a broader construct of uncontrolled eating. Additionally, individuals with de novo LOCE evidenced less frequent planned eating compared to those who never endorsed LOCE, suggesting that individuals at risk for LOCE development may be less likely to adhere to preoperative diet prescriptions. These individuals may benefit from longer and/or more intensive preoperative support involving cognitive-behavioral and behavioral change strategies, especially given that meal planning has been associated with better weight outcomes [18, 25, 26].

Those with maintained LOCE also reported less satiety sensitivity and greater hedonic hunger compared to those who never endorsed LOCE. Decreased satiety sensitivity and heightened hedonic eating may be key factors underlying LOCE maintenance over time, which is substantiated by research showing that individuals with binge eating show heightened responsiveness to food cues that promote eating in the absence of physiological hunger (Witt et al., 2014), coupled with deficiencies in the ability to detect homeostatic hunger and satiety cues [16, 27]. This may be further impacted by obesity given that BMI is inversely related to satiety sensitivity, and prior to surgery, an increased gastric capacity among individuals with obesity may have interfered with satiety signaling [28]. As such, patients who evidence lower satiety sensitivity and greater hedonic eating tendencies may benefit from interventions targeting interoceptive awareness and self-monitoring of hunger and fullness cues. For example, Regulation of Cues (RoC) therapy focuses on developing such skills, which has been associated with improvements in loss of control and overeating episodes among individuals with obesity [16]. In addition, increasing physical activity may be another way to improve satiety sensitivity and modulation of appetite, as our prior work has shown greater moderate-to-vigorous physical activity was associated with greater satiety in this sample [24].

Despite prior studies that have shown significant associations of LOCE before and after surgery with depressive symptoms, group status in this study was not significantly associated with preoperative depressive symptoms [2, 7, 10]. It may be possible that momentary negative affective states were more strongly associated with occurrences of LOCE in this sample, as studies have consistently demonstrated negative affect increases prior to binge-eating symptoms [29, 30]. Furthermore, preliminary findings suggest relationships between negative affect and LOCE may be stronger following surgery compared to before surgery [30]. It is of note that the mean preoperative BDI-II scores for all groups were within the minimal range (0–13) of symptom severity. Patients may have had greater positive affect during the preoperative period, potentially related to optimism and hopefulness regarding their treatment outcome.

Counter to expectations, restraint levels did not significantly differ between groups. There could have been some degree of social desirability bias that affected restraint ratings prior to surgery, with that, participants may have wanted to present themselves as attempting to restrain their eating in order to meet preoperative dietary guidelines. In addition, it is possible that there was significant heterogeneity in the nature of dietary restraint experienced within this sample prior to surgery, which may have contributed to inconsistent and non-significant differences between groups. That is, while some forms of restraint are adaptive and promote healthy weight management, rigid dieting has been implicated as a risk factor for binge eating and other disordered eating symptoms [17]. For example, one study performed by Ivazjez and colleagues found that individuals with BED reported significantly greater restraint than those who did not meet BED criteria but had either overweight or obesity [31]. The present study utilized adapted items from the cognitive restraint subscale of the Three-Factor Eating Questionnaire, and prior studies using this scale have shown variable associations between restraint, weight regulation, and binge-eating symptoms [32, 33]. Given that such relationships may depend on the type of restraint [34], it would be particularly useful for future studies of LOCE in bariatric samples to distinguish between rigid and flexible forms of dietary restraint.

It is important to note the limitations of this study. The sample size was modest and had attrition over the study period, which may have limited the ability to detect effects between groups, particularly the smallest group representing remitted LOCE. Yet, this remains the largest published study to date involving EMA of postoperative bariatric surgery patients. Nevertheless, it will be imperative for future studies to employ larger sample sizes across longer follow-up periods. Although analyses made use of all available EDE-Q postoperative data to characterize LOCE status, it is possible that analyses did not capture LOCE occurring outside of the 28-day time frame of this measure. The follow-up period was also limited to the initial 12 postoperative months, and it will be important for longer-term studies to examine LOCE course over time. In particular, given that the first postoperative year is typically the period of most pronounced weight loss, the effects of LOCE on weight outcomes might be delayed and manifest after weight has stabilized. The sample was predominantly female, and therefore, findings may not generalize to broader bariatric populations. Lastly, some potentially relevant factors (e.g., emotion regulation, shape and weight overvaluation) were not addressed in the current study that would be important to examine in future prospective studies of postoperative LOCE.

The current study adds to the growing body of literature documenting the importance of postoperative LOCE among bariatric surgery patients, and further highlights the need for long-term follow-up studies of LOCE course in this population. In particular, future research is needed to assess the longer-term impact of satiety sensitivity and hedonic eating on LOCE maintenance, as well as explore the extent to which meal planning and self-monitoring may buffer risk for the develop of LOCE following surgery. Addressing these factors prior to and/or after surgery may in turn support better mental health and weight loss outcomes.
